# Increased *Plasmodium falciparum* Parasitemia in Non-splenectomized *Saimiri sciureus* Monkeys Treated with Clodronate Liposomes

**DOI:** 10.3389/fcimb.2017.00408

**Published:** 2017-09-21

**Authors:** Janaiara A. Cunha, Leonardo J. M. Carvalho, Cesare Bianco-Junior, Márcia C. R. Andrade, Lilian R. Pratt-Riccio, Evelyn K. P. Riccio, Marcelo Pelajo-Machado, Igor J. da Silva, Pierre Druilhe, Cláudio Tadeu Daniel-Ribeiro

**Affiliations:** ^1^Laboratório de Pesquisa em Malária, Instituto Oswaldo Cruz, Fundação Oswaldo Cruz (Fiocruz) Rio de Janeiro, Brazil; ^2^Instituto de Ciência e Tecnologia em Biomodelos, Fiocruz Rio de Janeiro, Brazil; ^3^Laboratório de Patologia, Instituto Oswaldo Cruz, Fiocruz Rio de Janeiro, Brazil; ^4^Vac4All Initiative, Pepinière Paris Biotech Santé Paris, France

**Keywords:** malaria, *Plasmodium falciparum*, *Saimiri sciureus*, clodronate liposomes, macrophages, spleen, liver

## Abstract

A major constraint in the study of *Plasmodium falciparum* malaria, including vaccine development, lies on the parasite's strict human host specificity and therefore the shortage of animal experimental models able to harbor human plasmodia. The best experimental models are neo-tropical primates of the genus Saimiri and Aotus, but they require splenectomy to reduce innate defenses for achieving high and consistent parasitemias, an important limitation. Clodronate-liposomes (CL) have been successfully used to deplete monocytes/macrophages in several experimental models. We investigated whether a reduction in the numbers of phagocytic cells by CL would improve the development of *P. falciparum* parasitemia in non-splenectomized *Saimiri sciureus* monkeys. Depletion of *S. sciureus* splenocytes after *in vitro* incubation with CL was quantified using anti-CD14 antibodies and flow cytometry. Non-infected and *P. falciparum*-infected *S. sciureus* were injected intravenously twice a week with either CL at either 0.5 or 1 mL (5 mg/mL) or phosphate buffered saline (PBS). Animals were monitored during infection and treated with mefloquine. After treatment and euthanasia, spleen and liver were collected for histological analysis. *In vitro* CL depleted *S. sciureus* splenic monocyte/macrophage population in a dose- and time-dependent manner. *In vivo*, half of *P. falciparum*-infected *S. sciureus* treated with CL 0.5 mL, and two-thirds of those treated with CL 1 mL developed high parasitemias requiring mefloquine treatment, whereas all control animals were able to self-control parasitemia without the need for antimalarial treatment. CL-treated infected *S. sciureus* showed a marked decrease in the degree of splenomegaly despite higher parasitemias, compared to PBS-treated animals. Histological evidence of partial monocyte/macrophage depletion, decreased hemozoin phagocytosis and decreased iron recycling was observed in both the spleen and liver of CL-treated infected *S. sciureus*. CL is capable of promoting higher parasitemia in *P. falciparum*-infected *S. sciureus*, associated with evidence of partial macrophage depletion in the spleen and liver. Macrophage depletion by CL is therefore a practical and viable alternative to surgical splenectomy in this experimental model.

## Introduction

According to the World Health Organization (WHO) estimates, 212 million cases of malaria occurred in 2015, with 429,000 deaths (WHO, 2016). Among the *five* Plasmodium species capable of causing human malaria, *Plasmodium falciparum* is associated with more severe and lethal forms of the disease (Rowe et al., 2009; Quintero et al., 2011). Due to increasing parasite resistance to currently available antimalarial drugs (Chrubasik and Jacobson, 2010; Dondorp et al., 2010), development of an effective vaccine against malaria is urgently needed. Currently, *one* of the promising vaccine candidates (RTS,S/AS01) is being tested in a Phase III clinical trial in Africa in order to inform a decision regarding its deployment (Clemens and Moorthy, 2016; Olotu et al., 2016; Otieno et al., 2016). Another candidate, the MSP3 experimental vaccine, had a promising performance in African children in a double-blind follow-up of a preliminary Phase I study (Sirima et al., 2011). However, there is no assurance that these candidates will become indeed effective vaccines in the near future. Continued evaluation of potential vaccines is therefore expected and necessary.

Animal models for pre-clinical studies are an important component in vaccine development, but the limited availability of experimental models that can harbor human malaria is a critical constraint. The neo-tropical primates of the genus Saimiri and Aotus are experimental models recommended by WHO for pre-clinical testing of malaria vaccine candidates (WHO, 2004). Studies using the *Saimiri sciureus* model showed that immunization against *P. falciparum* blood stage vaccine candidate antigens such as the glutamate-rich protein (GLURP), the merozoite surface protein-3 (MSP3), or the SE36 antigen, using different adjuvants, may induce potent antibody responses and elicit partially protective immunity upon challenge (Carvalho et al., 2004, 2005; Tougan et al., 2013). Besides being susceptible to *P. vivax* and to *P. falciparum* infection (Gysin and Fandeur, 1983; Carvalho et al., 2000, 2002, 2004, 2005; Contamin et al., 2000; Herrera et al., 2002; Collins et al., 2005), these primates are abundant in nature and can be easily handled in captivity due to their relatively small size. Furthermore, they are able to reproduce clinical and pathological manifestations of human malaria such as thrombocytopenia, changes in leukocyte counts and anemia, offering an appropriate model to study the immunopathogenesis of malaria (Contamin et al., 2000; Carvalho et al., 2003). The limited availability of immunological tools to study the immune response in these animals imposes some constraints, but human reagents can be used to some extent and specific Saimiri reagents have been generated (Garraud et al., 1998; Contamin et al., 2005; Alves et al., 2010; Riccio et al., 2015). However, these models also have disadvantages. One of the major constraints is the need for splenectomy to achieve high and consistent parasitemias (Collins, 1992). The immune response against the erythrocytic forms of the parasite is largely mediated by resident cells in the spleen (Criswell et al., 1971; Achtman et al., 2003; Leisewitz et al., 2004) and this organ has critical roles in the immune response during malarial infections. Therefore, radical surgical splenectomy poses a strong limitation for testing malaria vaccines, as the very immune responses the vaccines are intended to elicit may be affected by the intervention. Therefore, alternative approaches to allow increased *P. falciparum* parasitemias in *S. sciureus* without the need for surgical splenectomy are necessary for proper evaluation of potential vaccines.

Liposomes containing a toxic chemical induce macrophage “suicide,” depleteing phagocytes in specific tissues. Clodronate, a bisphosphonate drug that activates apoptosis, has been the most widely employed compound in this respect. It has been demonstrated in several animal models, including mice, dogs, and pigs, that macrophages ingest the liposome particles by phagocytosis and are destroyed or become functionally inactivated (van Rooijen and van Nieuwmegen, 1984; Mathes et al., 2006; Kim et al., 2008). The spleen is a site of important macrophage activity and destruction of blood-borne microorganisms including Plasmodium, senescent red blood cells, and clodronate-encapsulated liposomes (van Rooijen and van Nieuwmegen, 1984; van Rooijen and Kors, 1989; van Rooijen et al., 1990; van Rooijen and van Kesteren-Hendrikx, 2002; Abbas et al., 2008). Chemical “splenectomy” by clodronate liposomes is expected to induce higher *P. falciparum* parasitemias as it depletes monocyte/macrophages in large numbers while preserving spleen structure and functions. Indeed, in immunocompromised mice lacking B, T, and NK cells, *P. falciparum* growth was fully controlled by macrophages. Conversely, *P. falciparum* could successfully replicate at very high parasite densities for several weeks when macrophage populations were controlled by repeated administration of clodronate-encapsulated liposomes (Badell et al., 2000; Arnold et al., 2010, 2011). If the same system can be used in *P. falciparum* infections in *S. sciureus* monkeys, it can provide a much improved model for testing malaria vaccines. In such a system, the phagocytic activity of monocytes/macrophages would be decreased allowing increased parasitemia in naive animals, but it is expected that effective vaccines inducing protective adaptative immune responses, in a scenario of preserved spleen structure and function, will be able to contain parasite growth.

In this study, we investigated whether a decrease in the number of monocytes/macrophages by using clodronate-encapsulated liposomes would favor the development of consistent *P. falciparum* parasitemias in *S. sciureus* monkeys without the need for surgical splenectomy, through a “chemical splenectomy.” This procedure would also be more respectful of ethical considerations than surgery, because of its transient nature resulting in fast restitutio ad integrum.

## Materials and methods

### Animals

The animals used in this study were male and female *S. sciureus*, karyotype 14-7, ranging in age from 3 to 19 years and in weight from 0.548 to 0.926 kg. The monkeys, non-splenectomized and malaria-naïve, were obtained from the breeding colony of the Primatology Service (CECAL/Fiocruz), Rio de Janeiro, Brazil. In total, 34 animals were used in *three* independent experiments (Table 1). This study was carried out in accordance with the recommendations and approved by the Fiocruz Ethics Committee on Animal Use (CEUA Licences L-0062/08 and LW-9/14).

**Table 1:** Number of *Saimiri sciureus* monkeys used per experiment.

	**Exp 1**	**Exp 2**	**Exp 3**	**Total**
NInf PBS	–	2	1	3
NInf 0.5 mL CL	–	2	1	3
NInf 1.0 mL CL	–	2	1	3
Inf PBS	3	2	3	8
Inf 0.5 mL CL	–	3	4	7
Inf 1.0 mL CL	3	3	4	10
TOTAL	6	14	14	34

*Exp, experiment; NInf, non-infected animals; Inf, P. falciparum-infected animals; CL, Clodronate-encapsulated liposomes*.

### Clodronate-encapsulated liposomes

Clodronate-encapsulated liposomes (CL, 5 mg/mL clodronate) were obtained from ClodronateLiposome.org (Department of Molecular Cell Biology and Immunology of the Vrije Universiteit Medisch Centrum - University Amsterdam, VUmc - Netherlands).

### Evaluation of clodronate-liposome on macrophages/monocytes *in Vitro*

Cryopreserved Saimiri splenocytes obtained during splenectomy of *three* naïve animals were thawed, washed twice with pure RPMI medium (Gibco, Grand Island, New York, EUA), resuspended in RPMI supplemented with 10% fetal bovine serum (FBS, Invitrogen, Grand Island, New York, EUA) at a concentration of 5 × 10^5^ cells/ 500 μL and incubated in 5 mL culture tubes (Falcon, BD Biosciences, San Jose, CA, EUA) in RPMI medium containing 15 mM glutamine (Gibco), 10 mM Hepes (Sigma-Aldrich, St. Louis, MO, EUA), 200 U/mL penicillin (Gibco), 200 μg/mL streptomycin (Gibco), 3 mg/mL gentamicin (Sigma-Aldrich) and 2 g/L sodium bicarbonate (Sigma-Aldrich) supplemented with 10% inactivated fetal bovine serum (Invitrogen). CL suspension was added at concentrations of 1, 10, and 100 μg/mL and incubated for 4, 8, 16, or 24 h at 37°C and 5% CO_2_. After culture, cells were labeled with anti-CD14-APC antibody (clone 61D3, eBioscience, Science Center Dr. San Diego, CA, EUA), a marker of macrophages and monocytes, and quantified by flow cytometry in the CyAn cell analyser (Dako Cytomation Inc., Carpinteria, CA, EUA) by counting 60,000 events. Data were analyzed using the software Summit (Dako Cytomation).

### Evaluation of clodronate-liposome *in Vivo*

*Plasmodium falciparum* FUP strain (Carvalho et al., 2004) was recovered from cryostabilates and the parasitized red blood cells (pRBCs) were passaged by intravenous inoculation in a splenectomized donor monkey. pRBCs from the passage animal were used to infect the experimental group animals intravenously with an inoculum of 10^6^ pRBC. The animals were divided into *six* groups, *three* with infected (Inf) animals and *three* control, non-infected (NInf) groups: (1) NInf receiving 1 mL of phosphate buffered saline (PBS - Sigma-Aldrich); (2) NInf receiving 0.5 mL of clodronate-encapsulated liposome (CL); (3) NInf receiving 1 mL of CL; (4) Inf receiving 1 mL of PBS; (5) Inf receiving 0.5 mL of CL; (6) Inf receiving 1 mL of CL. CL injections were performed intravenously *two* times a week, from day 0 of infection. *Three* independent experiments were performed using a total of 34 animals (Table 1).

The follow up of infection included a daily evaluation of parasitemia by thin Giemsa-stained blood films, hematocrit (microhematocrit method) and hemoglogin levels twice a week (using the HemoCue Hb301 system, HemoCue AB, Ängelholm, Sweden) as well as daily measurement of body (rectal) temperature. Blood monocyte count was done, twice a week starting from day 0 of infection, by flow cytometry using anti-CD14-APC antibody (clone 61D3, eBioscience) in a CyAn cell analyser (Dako Cytomation) and the data were analyzed using the software Summit (Dako Cytomation). An experienced veterinarian performed daily clinical examinations. Monkeys were treated with mefloquine (15 mg/kg—Biomanguinhos, Fiocruz, Rio de Janeiro, RJ, Brazil) when parasitemia reached 20% or above or in case the hematocrit reached 20% or below or when the monkeys presented manifestations of severe disease (prostration, anorexia). Monkeys that spontaneously controlled their parasitemia also received mefloquine on day 17 to ensure complete parasite clearance. *Four* days after mefloquine treatment, with animals no longer presenting patent parasitemia, all animals (including the uninfected controls) received a last injection of CL (or PBS) and were euthanized 24 h later. The animals were euthanized with 7 mg/kg thiopental (Cristália, Itapira, SP, Brazil) inoculated directly into the heart muscle, after being anesthesized with ketamine (Cristália) 100 mg/kg plus midazolam (União Química, Embu-Guaçu, SP, Brazil) 10 mg/kg. After the death of the animal, liver and spleen were harvested for histological analysis.

### Spleen and liver analysis

Immediately after liver and spleen harvest, macroscopic evaluation was performed and the spleen was weighted. Samples of the liver and spleen were fixed using a 4% formaldehyde solution (Merk - Darmstadt, Germany) in Millonig's buffer (0.1 M sodium hydroxide, 0.13 M sodium phosphate monobasic—Sigma-Aldrich) and later processed for histology. Paraffin-embedded sections (5 μm) were stained with hematoxylin and eosin (HE), Giemsa or Prussian blue. The slides were analyzed with the aid of a microscope (AxioImager A2, Zeiss, Oberkochen, Germany) and images were captured using an Axiocam HRM (Zeiss) and the software AxionVision Release 4.8.2 (Zeiss).

For hemozoin quantitation in the spleen, the histology slices were dyed with Nuclear Fast Red and mounted with coverslips. Each slide was then completely scanned using VSlide (Metasystems, Germany), generating high definition images (0.8 NA) of all the slices. Because the original images were too big, they were divided into *three* parts for analysis, by using ImageJ, where the red (nuclei) and brown/black (hemozoin) colors were separated with the color threshold function. The hemozoin areas and the total tissue area in each image were measured in square pixels. These results were then used to estimate the relative hemozoin area in each slide.

### Statistical analysis

The significance of the differences between the results or means of all variables was examined by the nonparametric Kruskal-Wallis analysis followed by Dunn test (GraphPad Prism 6, GraphPad Software Inc., San Diego, CA, USA). Results were considered to be statistically significant when *p* < 0.05.

## Results

### Effect of clodronate-encapsulated liposomes on *S. sciureus* monocytes *in Vitro*

**Figure 1:**
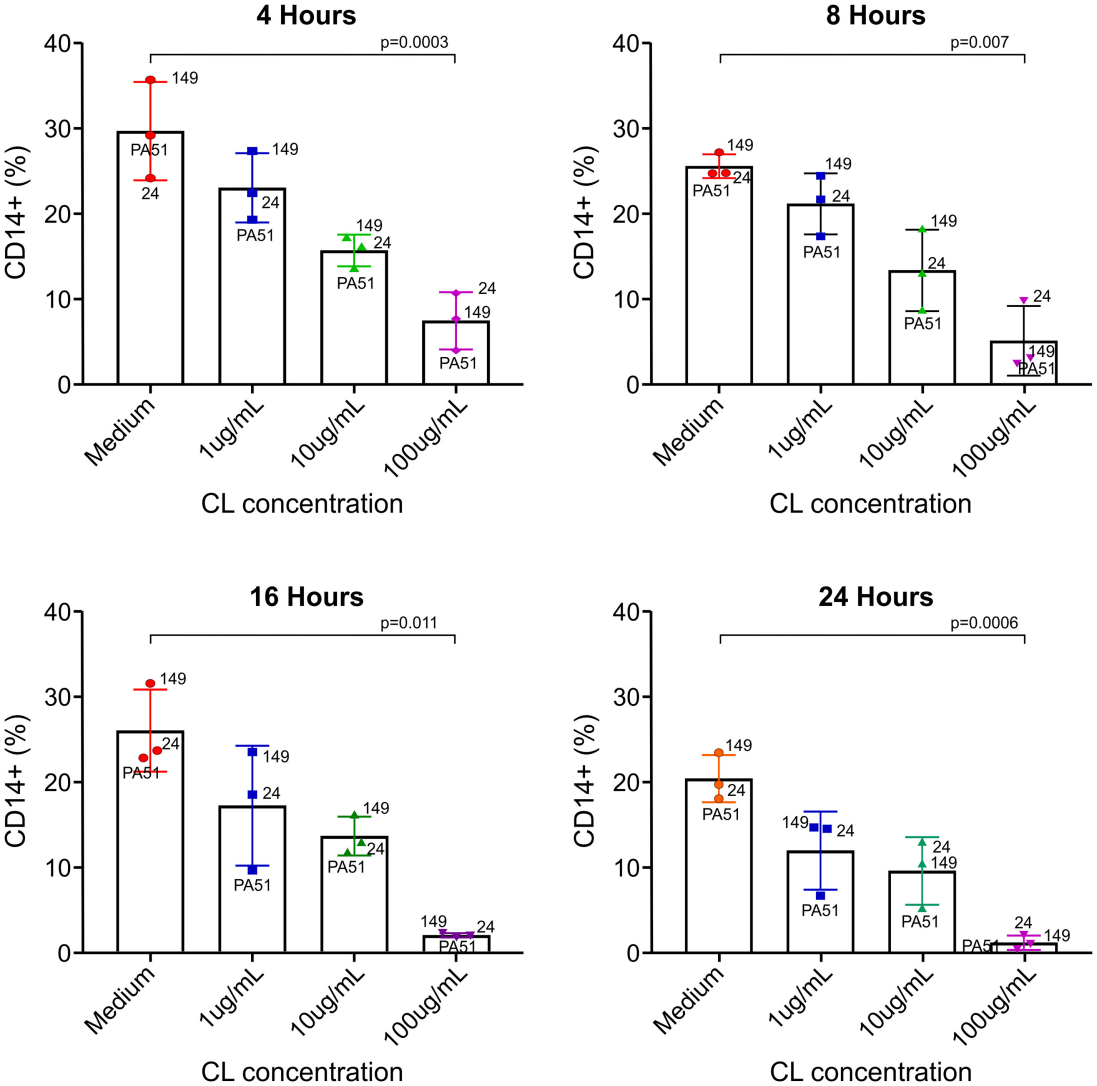
Effect of CL on *S. sciureus* splenocyte viability *in vitro*. CL induced dose-dependent cytotoxicity in monocytes/macrophages. 24, 149, and PA51 refer to *S. sciureus* identification. Medium vs. 100 μg/ml: 4 h (*p* = 0.0003), 8 h (*p* = 0.007), 16 h (*p* = 0.011), and 24 h (*p* = 0.0006). %: percentage of CD14+ cells in total number of splenocytes.

Even though CL has been used as a rapid inducer of apoptosis of monocytes/macrophages *in vivo* and *in vitro*, especially in mice, there are no available data on the effect of CL in *S. sciureus* monocytes. *In vitro* incubation of *S. sciureus* splenocytes with CL induced death of CD14+ cells (monocyte/macrophage) in a dose-dependent manner, with marked depletion at 100 μg/mL (Figure 1).

### Effect of clodronate-encapsulated liposomes on *P. falciparum* infection in *S. sciureus* monkeys

#### Parasitemia

**Figure 2:**
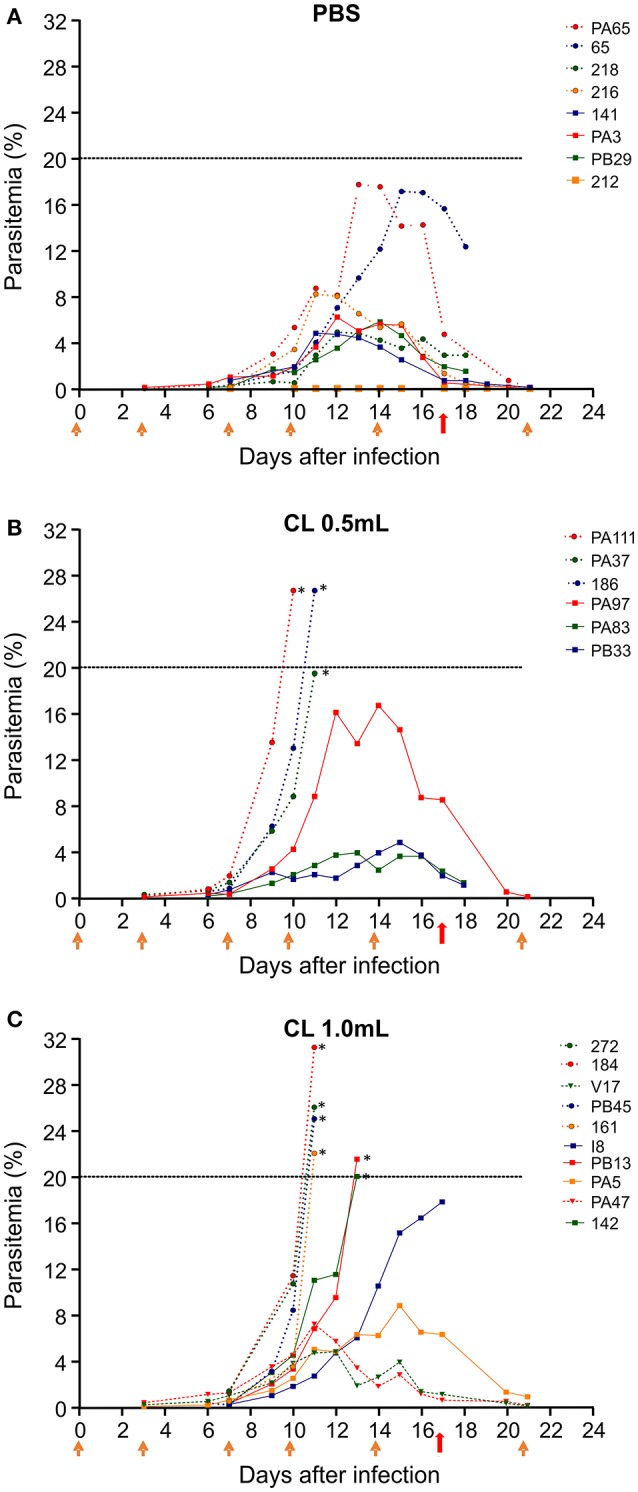
Course of parasitemia after infection of *S. sciureus* with 10^6^ pRBC/mL by FUP strain of *P. falciparum*. Parasitemia is shown for animals that received PBS (controls) **(A)**; clodronate liposomes (CL) 0.5 mL **(B)**; or CL 1.0 mL **(C)**. Data are from three separate experiments, with 6–9 animals per group. Orange arrows indicate the timepoints when PBS or CL was given to the animals. Red arrows indicate treatment with mefloquine. Dashed line represents the limit of parasitemia established for treatment (20%) with mefloquine. ^*^Animals that received treatment with mefloquine before day 17 for reaching 20% parasitemia.

*Saimiri sciureus* monkeys infected with *P. falciparum* and receiving PBS showed variable courses of parasitemia (Figure 2A). The maximum parasite density varied between 4.8 and 17.7% (peak average 8.1%). Most animals kept parasitemia below 10%, and one showed extremely low parasitemia throughout the follow up. The peak parasitemia was reached between days 11 and 15. All animals were able to control parasitemia without the need for antimalarial drug treatment. The infected animals receiving 0.5 mL CL also showed variable courses of parasitemia, which reached peak levels between days 10 and 15, with parasite densities ranging between 3.9 and 26.7% (average 16.4%) (Figure 2B). Of the *six* animals in this group, *three* (50%) required treatment with mefloquine due to high parasitemia (26.7, 19.5, and 26.7%). In the group of infected animals receiving 1 mL CL the maximum parasite density varied between 4.7 and 31.2% (average 18.5%) and peak parasitemia was reached between days 11 and 15. Of the *nine* animals in the group, *six* (66.6%) required antimalarial treatment due to high parasitemia (22; 26; 31.2; 25; 20, and 21.5%). Depending on the need for treatment and treatment time, animals received a minimum of *four* and a maximum of seven CL injections.

#### CD14+ cell count in peripheral blood

**Figure 3:**
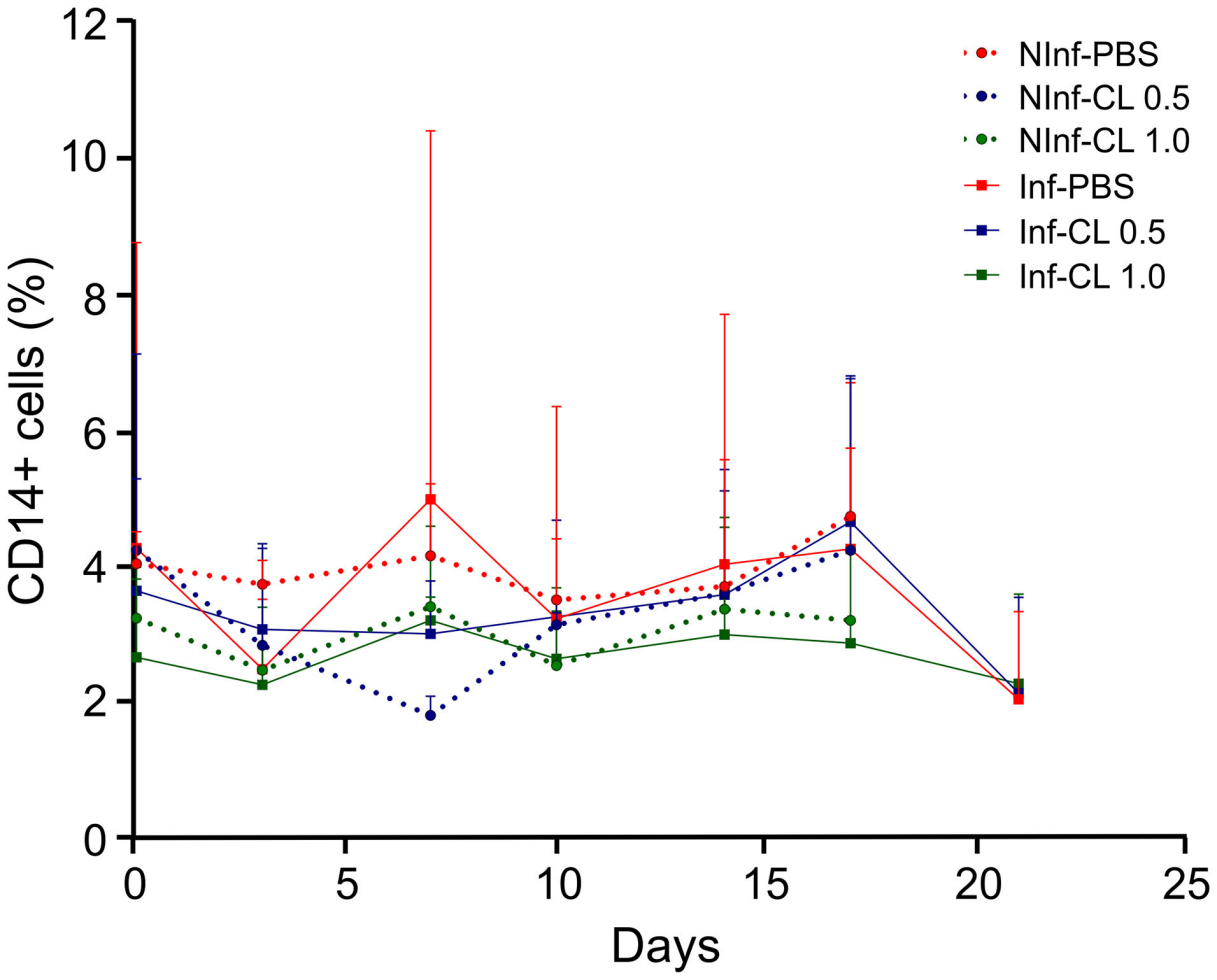
Changes in blood monocyte counts during *P. falciparum* infection and CL administration. Monocyte quantification was performed by flow cytometry using anti-CD14-APC antibodies. Solid lines represent the mean and standard deviation. %: percentage of CD14+ cells in total number of peripheral blood monocuclear cells (PBMC). NInf, non-infected control groups; Inf, *P*. *falciparum*-infected groups.

There was no apparent effect of *P. falciparum* infection or CL injections on circulating monocytes, as verified by CD14+ cell counts in peripheral blood (Figure 3).

#### Anemia and other clinical parameters

**Figure 4:**
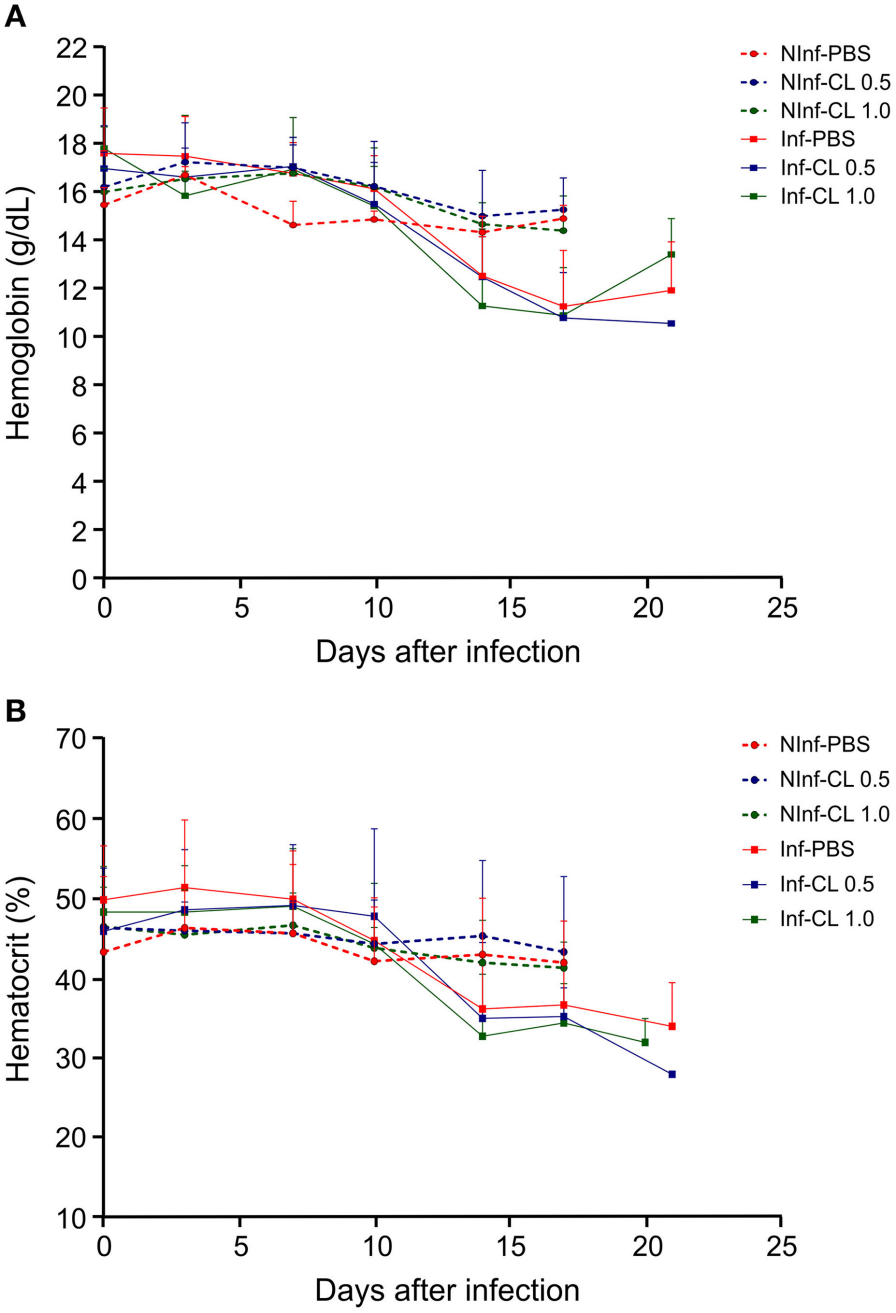
Hemoglobin concentration in the blood **(A)** and hematocrit **(B)** of *S. sciureus*. NInf, non-infected control groups; Inf, *P*. *falciparum*-infected groups. Solid lines represent the mean and standard deviation.

In *P. falciparum*-infected animals, hemoglobin concentration and hematocrit decreased reaching minimum values during or after parasite clearance (Figure 4). No weight loss greater than 10% was observed. Body temperature increased 1–2°C during infection and decreased after antimalarial treatment (data not shown). Some animals showed loss of appetite and prostration at the time of peak parasitemia. *Two* animals that received CL 1 mL and presented high parasitemias showed hematuria at the time of peak parasitemia that reversed after mefloquine treatment. Results of experiments 1, 2, and 3 showing the data of parasitemia, CD14 counting, hematocrit, hemoglobin and rectal temperature are shown in Supplemental Table 1.

#### Changes in the structure of the spleen and liver

All infected animals that developed parasitemia over 20% were treated with mefloquine between days 11 and 13 of infection, whereas all other infected animals were treated on day 17. All uninfected and infected-treated animals received a last injection of CL (or PBS) *four* days after mefloquine treatment, when parasitemia had cleared, and were euthanized 24 h later. Spleen and liver were harvested and processed for histological analysis.

##### Spleen

**Figure 5:**
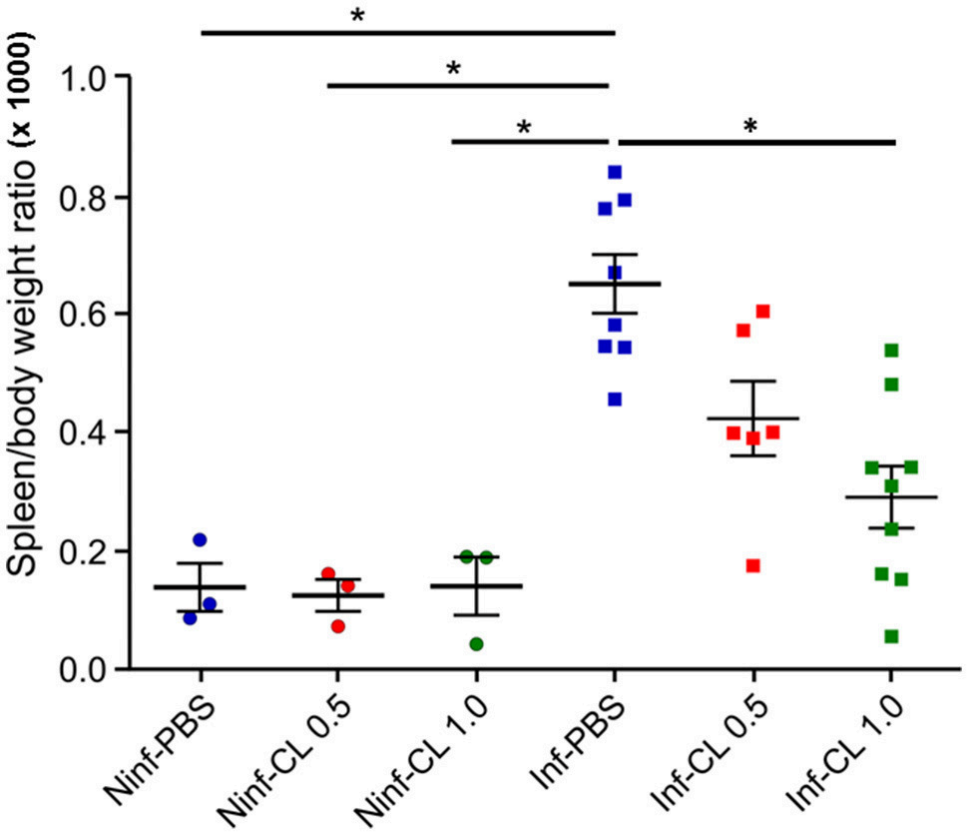
Effect of *P. falciparum* infection and/or CL administration on spleen/body weight ratios. Solid lines represent the mean and standard deviation. ^*^P < 0.05.

Treatment of uninfected *S. sciureus* with CL caused no perceivable changes in spleen weight in relation to uninfected animals that received PBS (Figure 5). *Plasmodium falciparum* infection, as expected, led to splenomegaly, with an average 5-fold increase in spleen/body weight ratios compared to uninfected animals. Treatment of *P. falciparum*-infected *S. sciureus* with CL 1 mL led to substantial reduction (average of 55%) in the degree of splenomegaly compared to infected animals that received only PBS (Figure 5 and Supplemental Table 1). The decrease in spleen/body weight ratios in animals treated with CL 0.5 mL was less prominent and did not reach significance compared to PBS-treated animals, although the lack of significance is likely due to the small sample size.

**Figure 6:**
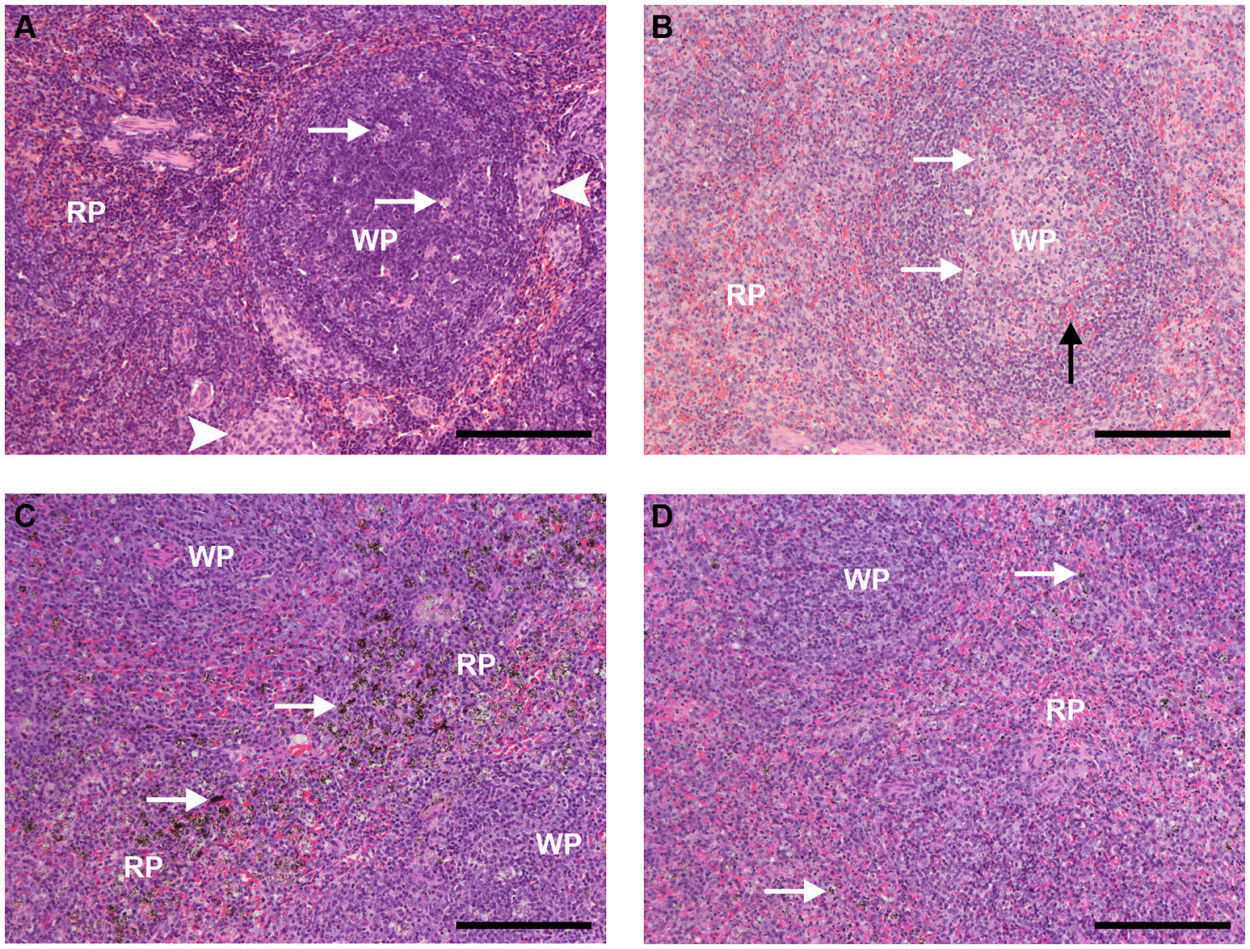
Spleen sections HE staining (bar: 200 μm). **(A)** Spleen of an uninfected, control animal that received PBS (code PA11). **(A)** B-cell follicle (WP, white pulp) is shown, with a few apoptotic centers (arrows); clusters of monocyte/macrophages, typical of Saimiri spleens, are present (arrowheads). The red pulp (RP) shows typical architecture, densily populated with small-nuclei, dark-stained cells, and red blood cells (red stain) flowing through. **(B)** Spleen of an uninfected Saimiri inoculated with CL 1 mL (code V3). In general, the splenic structure was preserved, but cell density was apparently smaller as compared to animals that received PBS, with predominance of cells with light-stained nuclei; penetration of red blood cells inside B-cell follicle was observed (black arrow); apoptotic centers (arrows), which are dependent on macrophages, and clusters of monocyte/macrophages were decreased (clusters of monocytes absent in this image). **(C)** Spleen of a *P. falciparum*-infected Saimiri that received PBS (code 141: treated at day 17, with 0.69% parasitemia, killed 5 days later). Emphasis is given to the distribution of hemozoin (seen as dark, granulated stain - arrows), which could be found throughout the red pulp (RP); loss of well-defined limits between the RP and the white pulp (WP) was observed. **(D)** Spleen of a *P. falciparum*-infected Saimiri that received CL 1 mL (code 272: treated at day 11, with 22 % parasitemia, killed 5 days later). The amount of hemozoin (arrows) was substantially decreased compared to infected animals that received PBS, and seen as small black dots. As in **(C)**, loss of well-defined limits between the red pulp (RP) and the white pulp (WP) was observed.

The spleen of uninfected, control animals that received PBS showed typical *S. sciureus* splenic architecture, with well-defined limits between the red and white pulps, resting T cell areas, mostly resting follicles, and in some cases with phase I and phase II germinal centers containing a few apoptotic centers, absence of pigment and plasmacytes, and areas of monocyte accumulation in the marginal zone and the red pulp (Figure 6A), as previously described (Alves et al., 2015). Uninfected animals that received CL (0.5 or 1 mL) showed decreased number of clusters of monocyte/macrophages in red pulp and of apoptotic centers in the follicles compared to animals that received PBS (Figure 6B).

Infected animals that received PBS showed phagocitosed malarial pigment (hemozoin) throughout the red pulp (Figure 6C). Erythroid precursors and plasmacytes were observed, and the marginal zone was in disarray (Figure 6C). Infected animals that received CL 1 mL showed substantial reduction in the accumulation of malarial pigment in the red pulp (Figure 6D), despite the fact that these animals in general showed much higher parasitemias (average 18.5%) as compared to infected animals that received PBS (average 8.1%), although for shorter periods of time (Figure 2).

**Figure 7:**
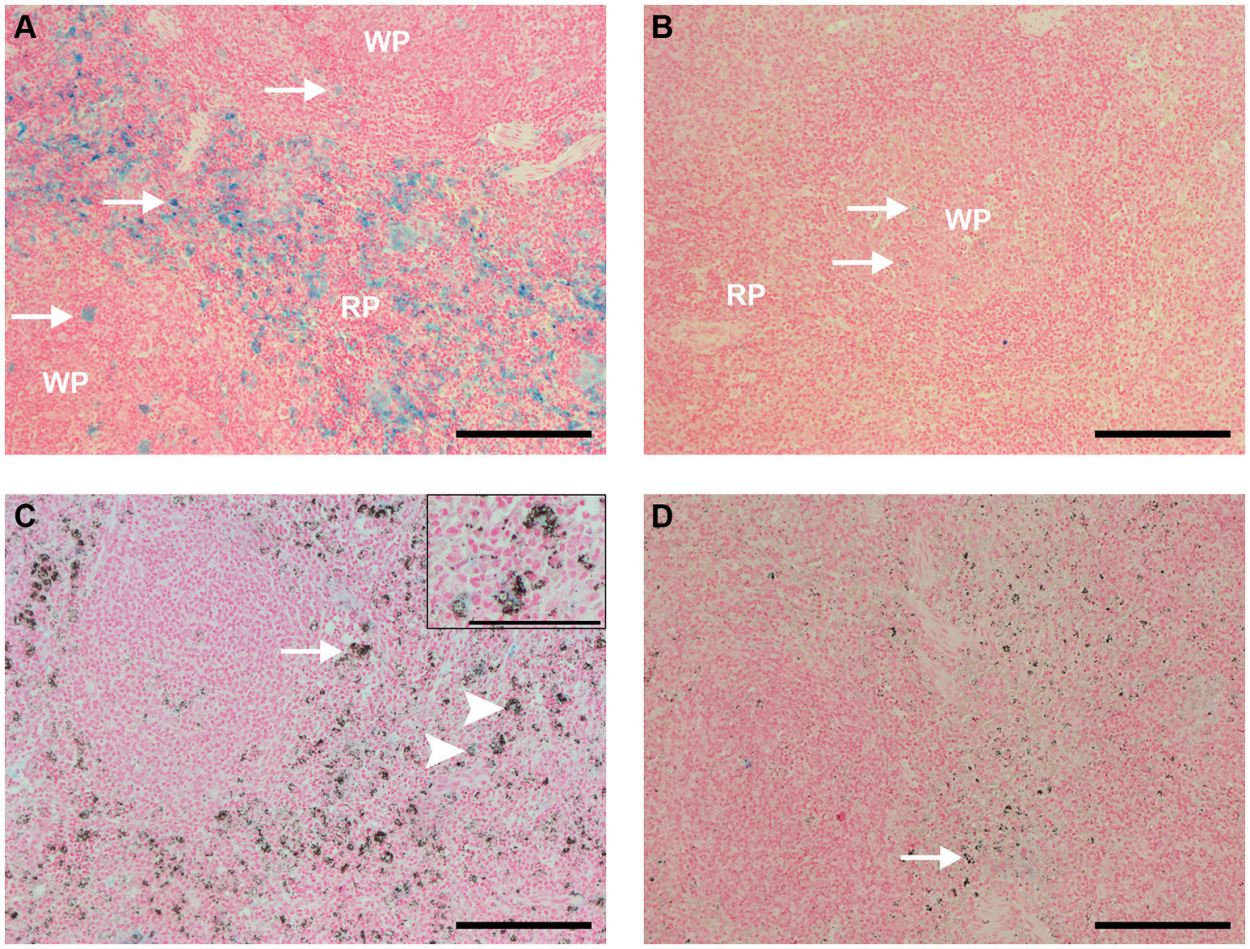
Spleen sections Perls staining (bar: 200 μm, except in **C**, bar: 100 μm). **(A)** Spleen of an uninfected, control animal that received PBS (code PA11). Large amounts of ferric iron-containing macrophages (blue staining) were observed throughout the red pulp (RP), and also sparsed in follicles (arrows). **(B)** Spleen of an uninfected Saimiri inoculated with CL 1 mL (code PB75). Blue staining, representing ferric iron-containing macrophages, nearly disappeared from the red pulp (RP), and was faint in follicles (arrows). **(C)** Spleen of a *P. falciparum*-infected Saimiri that received PBS (code 216: treated at day 17, with 1,3% parasitemia, killed 5 days later). Blue staining, representing ferric iron-containing macrophages, was much fainter in the spleen of infected, compared to uninfected, *S. sciureus*; on the other hand, macrophages were laden with hemozoin (arrow); only few macrophages showed colocalization of iron staining and hemozoin (arrowheads and magnified insert on top right). **(D)** Spleen of a *P. falciparum*-infected Saimiri that received CL 1 mL (code 272: treated at day 11, with 22% parasitemia, killed 5 days later). Presence of iron staining was nearly absent, and presence of hemozoin (arrow) was much decreased in relation to **(C)**.

The analysis of spleen sections stained with Perls, which shows ferric iron (Fe^3+^) within macrophages and can be used not only to observe the physiology of iron recycling but also to estimate the distribution of these cells, revealed that uninfected, control animals showed clusters of iron-containing macrophages distributed throughout the red pulp, and mild staining within follicles (Figure 7A). Uninfected, control animals that received CL 0.5 and 1 mL showed reduced amounts of iron-containing macrophages (Figure 7B).

Infected animals treated with PBS showed iron staining in the red pulp, but substantially less than uninfected controls (Figure 7C). However, as mentioned above (Figure 6C), large numbers of hemozoin-containing macrophages were observed. As a rule, there was little or absent colocalization of iron staining and hemozoin (macrophages showed only *one* staining, Figure 7C). Infected animals treated with CL showed less iron staining as compared to animals that received PBS, and also less hemozoin staining (Figures 7D, **10**).

##### Liver

**Figure 8:**
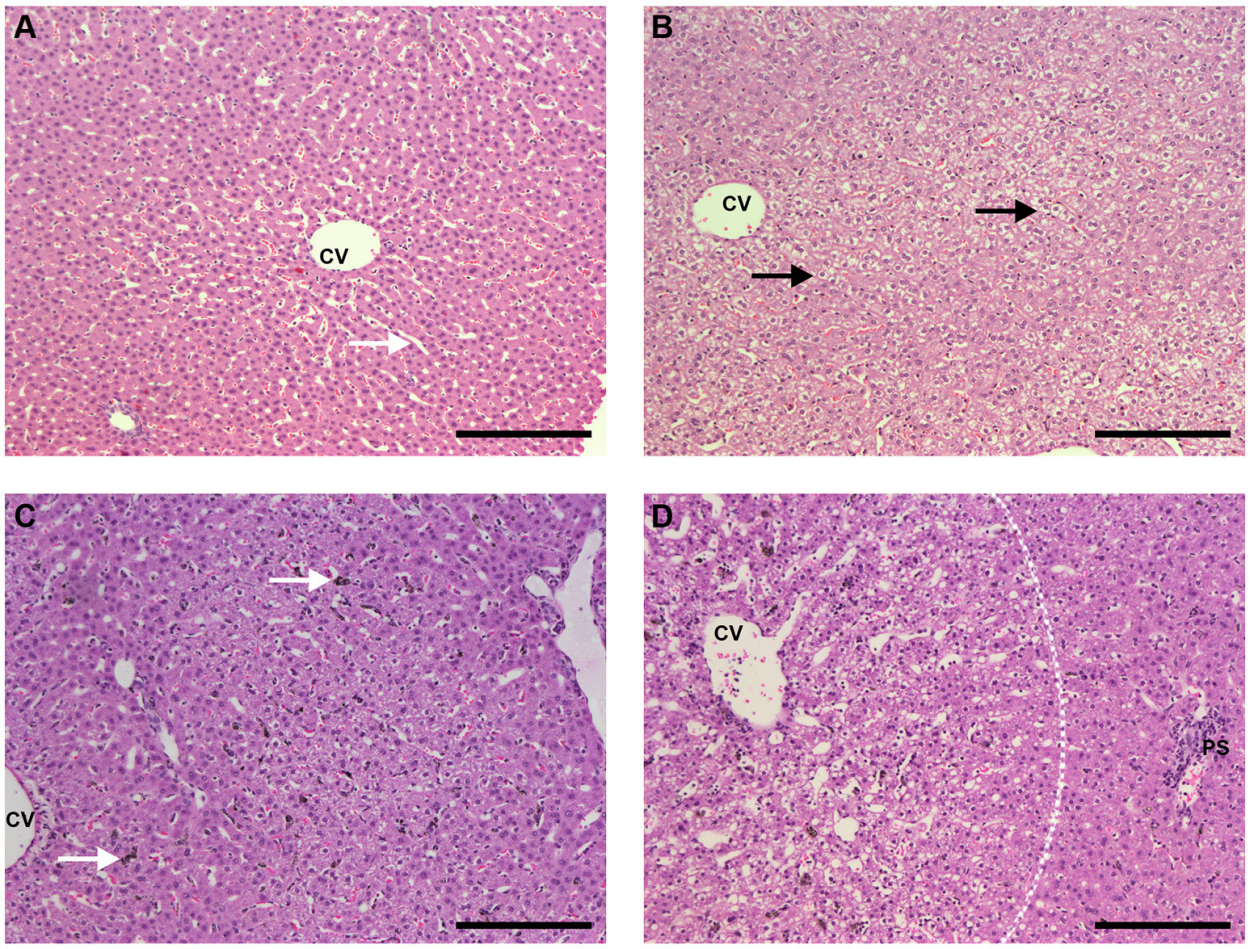
Liver sections HE staining (bar: 200 μm). **(A)** Liver of an uninfected, control animal that received PBS (code PA67), showing typical architecture, healthy hepatocytes and clean synusoids (arrows); CV, centrilobular vein. **(B)** Liver of an uninfected Saimiri inoculated with CL 1 mL (code PB75). The general aspect of most sections was rather normal (similar to the pattern of **A**), but eventually areas of hepatocyte vacuolization, as depicted, were observed; CV, centrilobular vein. **(C)** Liver of a *P. falciparum*-infected Saimiri that received PBS (code 141: treated at day 17, with 0.69 % parasitemia, killed 5 days later). Kupffer cells laden with hemozoin (arrow); CV, centrilobular vein. **(D)** Liver of a *P. falciparum*-infected Saimiri that received CL 1 mL (code 161: treated at day 11, with 22% parasitemia, killed 5 days later). Intense hepatocyte vacuolization was observed, evidenced particularly in the left half of the image, closer to the centrilobular vein (CV) and away from the portal space (PS) (more vacuolized area and more preserved area separated by a dashed line).

With HE staining, livers of uninfected control animals that received PBS showed typical structure, with healthy hepatocytes and absence of necrosis or vacuolization and Kupffer cells without pigment (Figure 8A and Supplemental Figure 1A). Mononuclear cell infiltrates in the portal space were occasionally observed. Livers of animals that received CL 0.5 or 1 mL showed either no significant changes or, in some cases, evidence of hepatocyte vacuolization. Figure 8B shows *one* of these areas of hepatocyte vacuolization, an event that was only occasionally found but may indicate some degree of toxicity induced by CL treatment.

Infected animals that received PBS showed large numbers of mononuclear cells and also erythroid cells, neutrophils and plasmacytes within sinusoids, mononuclear cell infiltration in the portal space, and Kupffer cells containing malarial pigment (Figure 8C and Supplemental Figures 1B–D). Diffuse vacuolization of hepatocytes was observed in *two* thirds of the animals. Infected animals that received CL showed areas of intense hepatocyte vacuolization, and there were apparently fewer Kupffer cells with malarial pigment than in infected animals that received PBS (Figure 8D).

**Figure 9:**
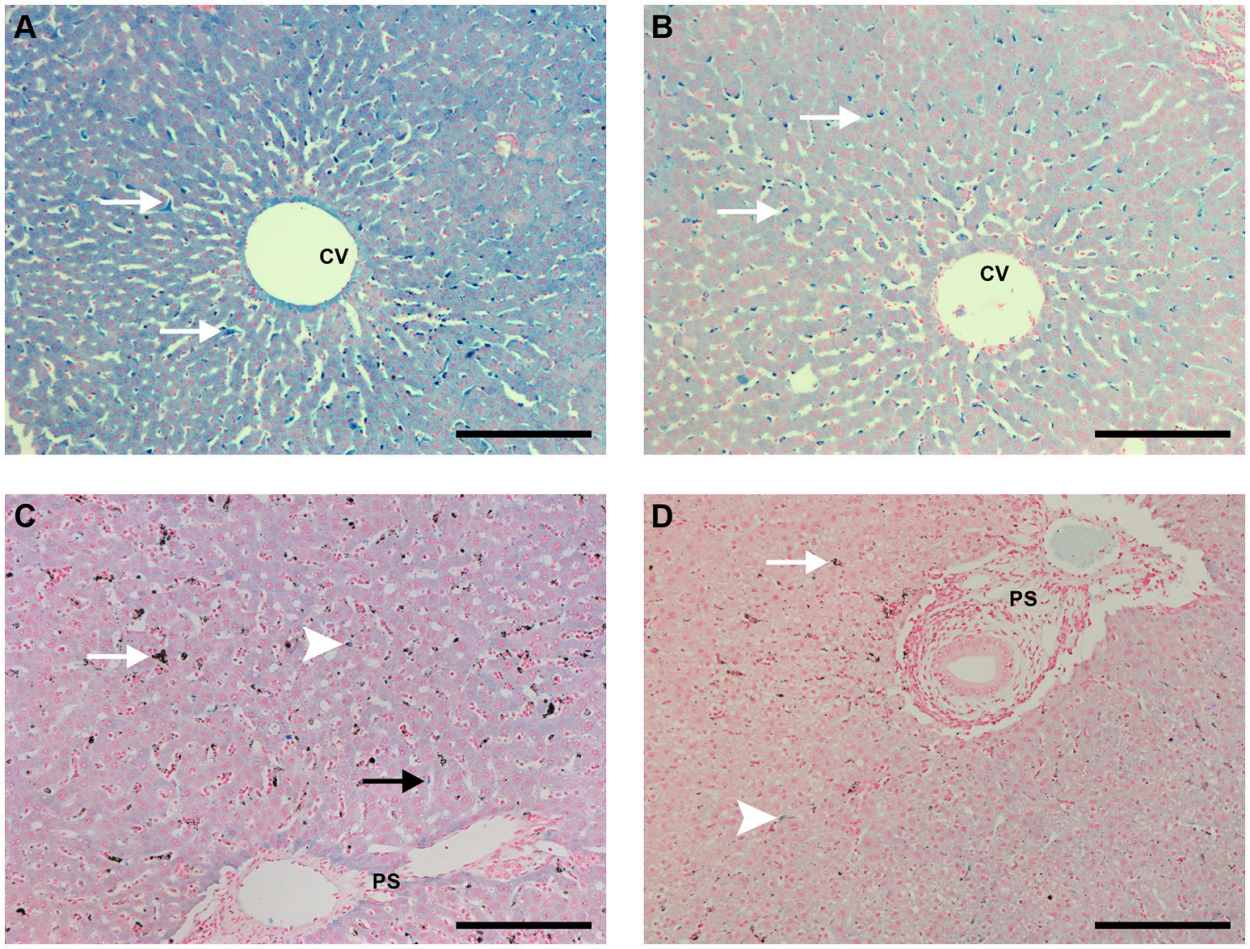
Liver sections Perls staining (bar: 200 μm). **(A)** Liver of an uninfected, control animal that received PBS (code PA11), showing blue staining throughout the hepatocytes, with stronger staining in Kupffer cells (arrows); CV: centrilobular vein **(B)** Liver of an uninfected Saimiri inoculated with CL 1 mL (code V3). The blue stainingwas lighter, especially in hepatocytes, with Kupffer cells still showing more pronounced staining (arrows). CV, centrilobular vein. **(C)** Liver of a *P. falciparum*-infected Saimiri that received PBS (code 216: treated at day 17, with 1,3% parasitemia, killed 5 days later). Iron staining was lighter than **(A)** in both hepatocytes and Kupffer cells, but still visible; Kupffer cells were laden with hemozoin (arrow); in only sporadic cases colocalization of hemozoin and iron staining was observed in Kupffer cells (arrowhead). PS: portal space. **(D)** Liver of a *P. falciparum*-infected Saimiri that received CL 1 mL (code 161: treated at day 11, with 22% parasitemia, killed 5 days later). Both iron staining (arrowhead) and hemozoin (arrow) were decreased in relation to **(C)**. PS: portal space.

Perls staining of liver sections revealed that uninfected animals that received PBS showed strong iron staining throughout the organ, with granules within hepatocytes and Kupffer cells (Figure 9A). Iron staining in liver sections of uninfected animals that received CL 0.5 or 1 mL was less intense than in those that received PBS (Figure 9B).

Infected animals that received PBS showed large amounts of hemozoin. Iron staining was weak throughout the organ, but still present in hepatocytes and isolated Kupffer cells or adherent macrophages within sinusoids, with little or no colocalization with hemozoin (Figure 9C and Supplemental Figures 2A,B). Infected animals that received CL 0.5 or 1 mL showed less intense hemozoin and iron staining, and Kupffer cells or macrophages with strong iron staining were rarely observed or not at all (Figure 9D).

#### Estimating the efficacy of CL treatment in depleting macrophages through splenic hemozoin quantification

**Figure 10:**
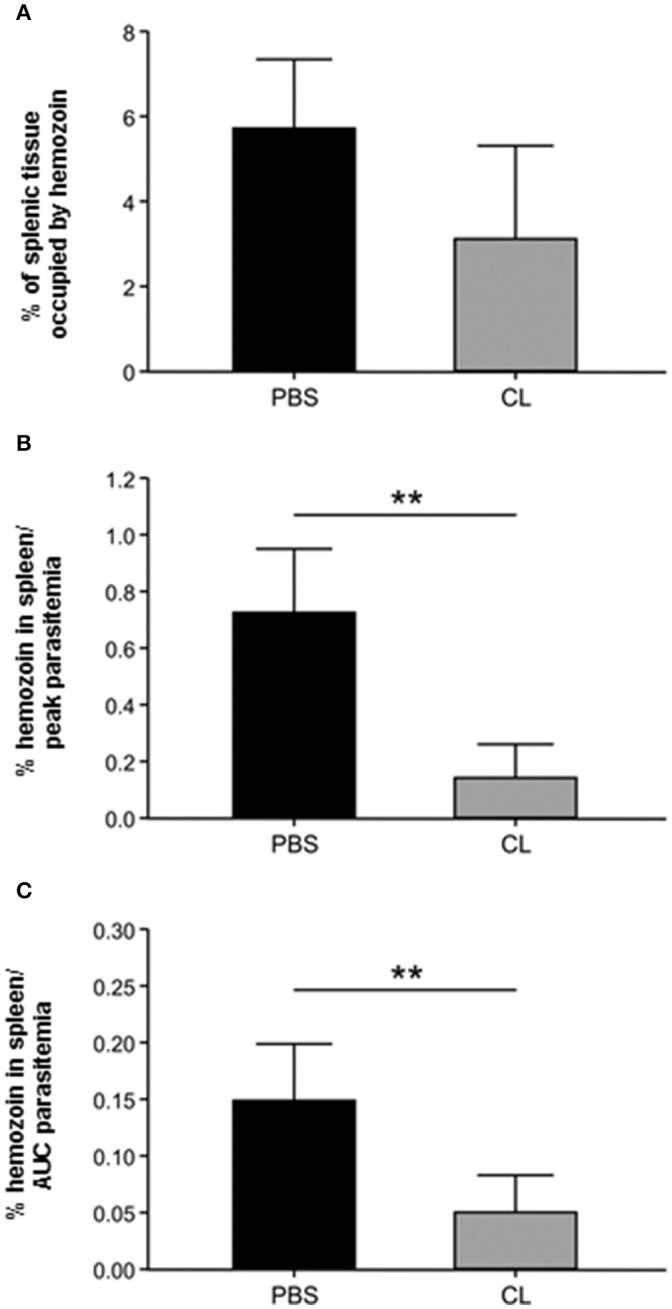
Estimation of the effect of CL treatment on splenic macrophage population through hemozoin quantification. **(A)** Percentage of the splenic tissue occupied by hemozoin in PBS-treated and CL-treated infected animals. **(B,C)** Amount of splenic hemozoin adjusted by parasitemia in PBS-treated and CL-treated infected animals. The percentage of splenic tissue occupied by hemozoin (as in **A**) was divided by the peak parasitemia **(B)** or by the area under the curve (AUC) of parasitemia **(C)** of each animal. ^**^P < 0.01.

Qualitative analysis of spleen and liver, as shown in Figures 6–9, and decreased spleen/body weight ratios (Figure 5) indicated that CL treatment led to a decrease in macrophage population in these organs. Attempts to quantify macrophages using immunostaining with anti-human CD14, CD68, and HAM-56 monoclonal antibodies were unsucessful, probably due to the prolonged period in formalin. The effect of CL treatment on splenic macrophage numbers was therefore further estimated by quantifying hemozoin accumulation in this organ, which increases with increasing parasitemia (Sullivan et al., 1996), and hemozoin-laden macrophages remain in the spleen for several weeks (Frita et al., 2012). Although, CL-treated monkeys showed about half the splenic hemozoin accumulation as compared to PBS-treated animals (Figure 10A), this difference did not reach statistical significance due to small sample size and to some degree of variation between animals. However, when hemozoin concentration was adjusted to the level of parasitemia (hemozoin concentration/peak parasitemia, Figure 10B; hemozoin concentration/area under the curve of parasitemia, Figure 10C), marked differences were observed between the *two* groups, indicating that despite sustaining much higher parasite loads CL-treated animals showed less hemozoin accumulation in the spleen. These data indicate that splenic macrophages of CL-treated animals are either markedly decreased in numbers, as also suggested by HE staining and spleen weights, or are functionally impaired, in any case resulting in decreased capacity to phagocytose parasitized red blood cells.

## Discussion

Saimiri monkeys are susceptible to infection by *Plasmodium falciparum* and, together with Aotus, constitute unique experimental models for malaria (WHO, 2004), making these animals particularly useful in preclinical trials of potential malaria vaccines and for studies on pathogenesis (Carvalho et al., 2000, 2004, 2005). Pre-clinical evaluation of vaccines in these experimental models can provide valuable information about the immunogenicity, efficacy and safety of a variety of formulations, facilitating selection for humans trials. Since access to this valuable resource is restricted, with limited number of animals available for research, optimization of the model is needed to allow the design of experiments with reduced sample sizes. This goal faces *two* important limitations regarding the use of non-splenectomized animals: (1) they usually develop low parasitemias, which makes it more difficult to detect the effect of interventions, such as vaccines, on the course of infection; and (2) there is substantial interindividual variability in the levels of parasitemias (Contamin et al., 2000). These *two* factors underscore the need to increase sample sizes in experiments using these animals. The alternative to overcome these limitations has been the use of splenectomized animals, which achieve higher and more consistent parasitemias, allowing smaller experimental groups to be formed. However, this strategy also faces important setbacks: (a) the spleen plays a central role in immunity against plasmodial infections and (b) non-human primates (NHP) are chosen as models for experimental studies with human plasmodia, mainly vaccines, because of their close filogenetic relationship to man. Splenectomy pushes back the model from the natural human situation. For these reasons, splenectomy constitutes a severe handicap especially in immunity and vaccine studies. The use of the “chemical (temporary) splenectomy” described here offers therefore an animal model closer to the target population condition.

In plasmodial infections, the spleen helps to control parasite burden through innate and adaptative immune responses (Yazdani et al., 2006; Portillo et al., 2012; Gazzinelli et al., 2014). Splenic macrophages are major players in this system, clearing parasitized red blood cells through phagocytic activity. Because *S. sciureus* are not *P. falciparum's* natural host, splenic clearance seems to be particularly effective, preventing higher parasitemias. We asked whether partial depletion of the macrophage population in the spleen and other sites would result in higher and more consistent parasitemias in *S. sciureus* while maintaining not only the structure but also the function of the spleen. Together, our results indicate that this goal was largely achieved in this study.

*Saimiri sciureus* monkeys infected with *Plasmodium falciparum* and treated with periodic intravenous injections of clodronate-encapsulated liposomes (CL) in general developed higher parasitemias requiring antimalarial treatment than infected monkeys treated with PBS. It is not clear why this effect was not observed in *three* out of 9 (33.3%) infected *S. sciureus* treated with CL 1 mL, as they were able to self-control parasitemia, behaving closer to PBS-treated animals. *Three* independent experiments were performed, and these *three* animals were from the same experiment, suggesting that the lot of CL used may not have been optimal. Alternatively, this result would confirm that there is some degree of individual animal variation, either in the efficacy of CL treatment or in the pre-existing state of non-adaptive immunity. It is likely that the number of macrophages will vary from *one* animal to the other. It is advisable that each lot of CL be assayed *in vitro* for monocyte killing capacity prior to the conduction of *in vivo* experiments.

In any case, the efficacy of CL 1 mL treatment was evident, as *two* thirds (6 out of 9) of the treated animals developed parasitemias over 20% requiring mefloquine treatment, whereas all *eight* PBS-treated animals self-controled their parasitemias. Administration of a smaller amount of CL (0.5 mL) also resulted in higher parasitemias overall, with half (3 out of 6) of the animals showing parasitemias over 20%. Results are in agreement with those recorded with *P. falciparum* in immunocompromised mice grafted with human RBCs (Badell et al., 2000; Arnold et al., 2010). Thus, it is very likely that higher CL doses would result in improved macrophage control, and thereby in higher parasitemias. This remains to be investigated in future experiments.

From an ethical point of view CL treatment offers the paramount advantage of avoiding heavy surgery and allowing full recovery of animals. It thus constitutes a major improvement from a scientific as well as from an ethical viewpoint over splenectomy.

*Plasmodium falciparum* infection and/or CL treatment had little effect on peripheral blood monocyte counts, which is not a surprise as young monocytes have limited phagocytic activity and CL targets preferentially large and active macrophages, which ingest a larger number of liposomes per cell. Indeed, CL injections resulted in marked effects in the spleen and the liver. In uninfected animals, no evident effect of CL injections was observed in spleen/body weight ratios, but the number and distribution of ferric iron-containing cells in the spleen were markedly reduced. Macrophages are involved in senescent red blood cell phagocytosis and iron recycling, and therefore CL injections largely depleted this cell population in the spleen. In *Plasmodium falciparum*-infected animals, CL injections resulted in milder splenomegaly and this effect was associated histologically with marked reductions in hemozoin-containing cells and further reductions in iron-containing cells. Efforts to further substantiate the histological findings by specific macrophage staining with commercially available anti-human CD14, CD68, and HAM-56 monoclonal antibodies were unsucessful, probably due to the prolonged period the biological material was kept in formalin.

Our results also indicate that *P. falciparum* infection in *S. sciureus* causes impaired iron-recycling by macrophages. Infected animals showed large numbers of hemozoin-containing macrophages but decreased numbers of ferric iron-containing cells in the spleen. An effect on iron homeostasis was also observed in the liver. It is known that macrophages are very important actors in controlling the iron upload and metabolism in hepatocytes, mostly via hepcidin. Therefore, it is possible that the reduction in iron staining in hepatocytes after CL treatment resulted from the instability of the hepcidin production due to Kupffer cell destruction (Fleming, 2005; Makui et al., 2005). However, additional studies are needed in order to clarify the mechanisms behind this phenomenon. In addition, colocalization of hemozoin and iron was a rare event, indicating that phagocytes were busy processing hemozoin from infected red blood cells and their capacity to carry out phagocytosis of uninfected red blood cells was impaired. Although the dark and gross pattern of hemozoin might actually prevent visualization of the light blue iron staining and therefore colocalization might be underestimated, some facts suggest that iron uptake by macrophages was impaired: (i) even macrophages with little, sparse amount of hemozoin showed no iron staining; (ii) colocalization was observed in some cells heavily laden with hemozoin. Since it has been shown that hemozoin may persist for weeks to months after parasite clearance (Frita et al., 2012; Alves et al., 2015), it may negatively impact the immune responses and iron recycling of the affected individuals for long periods of time after treatment has been implemented and therefore, contribute significantly to increasing malaria morbidity. Interestingly, in infected animals, Kupffer cells and sinusoidal macrophages heavily laden with iron were observed. This finding may indicate that young cells from the bone marrow repopulate the liver in an effort to restore iron recycling and other functions.

Depletion of macrophages by CL shows a number of advantages over surgical splenectomy for the study of malaria vaccines in Saimiri monkeys. However, macrophages are very important in the immune responses against *P. falciparum* through direct infected red blood cell phagocytosis or antibody-mediated parasite opsonization or inhibition (Bouharoun-Tayoun et al., 1990; Buffet et al., 2011). Therefore, sharp decreases in macrophage numbers might affect the antiplasmodial immune responses a given pre-clinical vaccine trial may be attempting to evaluate. The same limitation can be considered for dendritic cells, which also play relevant roles in antiplasmodial immunity (Urban et al., 1999, 2001). Indeed, phagocytic marginal dendritic cells but not interdigitating dendritic cells are known to be depleted by CL treatment (Leenen et al., 1998), which can affect immune responses to particulate antigens (Delemarre et al., 1990). These limitations need to be taken into account when using CL treatment as the strategy to induce higher *P. falciparum* parasitemias.

It has been shown that plasmodial infections in humans (Urban et al., 2005), Saimiri (Alves et al., 2015) and mice (Achtman et al., 2003; Carvalho et al., 2007; Martins et al., 2009) induce disarray of the spleen and other lymphoid organs, with disturbance of germinal center architecture. This finding was confirmed in the present study in *S. sciureus*, and CL administration had no apparent effect on this event. The splenic changes observed in *P. falciparum*-infected, saline-treated Saimiri 5 days after mefloquine treatment were similar to those recently reported for *P. falciparum*-infected Saimiri at peak parasitemia before antimalarial treatment (Alves et al., 2015). These changes included the presence of large numbers of phagocytes in the red pulp heavily laden with malaria pigment, disarray of B-cell follicles, blurred limits between the red and white pulps, and penetration of RBCs into follicles. In that study, 14 days after chloroquine treatment, spleens showed better white pulp organization and persistence of hemozoin in the red pulp but with a different pattern with more compacted, less granulous pigment (Alves et al., 2015). Therefore, the pattern of splenic changes observed in infected animals 5 days after mefloquine treatment in the present study was more similar to the changes observed at peak parasitemia prior to antimalarial treatment than after an extended period (2 weeks) after antimalarial treatment. This finding suggests that 5 days following antimalarial drug treatment is insufficient to reverse the pathological changes in spleen structure induced by *P. falciparum* infection.

Finally, infected animals that received CL showed more intense hepatocyte vacuolization. This effect was most likely a result of a combination of CL injection with higher parasitemias in these animals, because both uninfected animals receiving CL and infected animals receiving PBS showed some degree of hepatocyte vacuolization, milder than when the events were combined.

In conclusion, these results indicate that CL depleted splenic macrophages leading to decreased parasite phagocytosis, decreased splenic congestion, and increased parasitemias. CL administration is therefore a practical and viable alternative to surgical splenectomy in this experimental model.

## Author contributions

JC participated in study design, carried out the experiments and helped LC in drafting the manuscript; CBJ participated in study design and carried out the experiments, MA, LP-R, ER, and MP carried out the experiments and reviewed the manuscript; IdS carried out the experiments; LC, CD-R, and PD conceived the study, participated in its design and coordination, and reviewed the manuscript. All authors have read and approved the final manuscript.

### Conflict of interest statement

The authors declare that the research was conducted in the absence of any commercial or financial relationships that could be construed as a potential conflict of interest.

## Supplementary material

Supplemental Figure 1 Liver sections, HE (bar: 100 μm). **(A)** Liver of an uninfected, control animal that received PBS (code PA67), in higher magnification than Figure 8A, showing Kupffer cells (arrows). **(B–D)** Liver of a *P. falciparum*-infected Saimiri that received PBS (code 141: treated at day 17, with 0.69% parasitemia, killed 5 days later), in greater detail compared to Figure 8C. Portal infiltrates (black arrow), increased cellularity in sinusoids with presence of mononuclear cells (white arrowhead), erythroid cells (thin arrow) and plasma cells (black arrowhead).

Supplemental Figure 2 Liver sections, Perls (bar: 100 μm). **(A,B)** Liver of a *P. falciparum*-infected Saimiri that received PBS (code 216: treated at day 17, with 1.3% parasitemia, killed 5 days later), in greater detail compared to Figure 9C. Intrasinusoidal macrophages, circulating or attached to the sinusoidal wall, laden with malarial pigment (white arrows). There was little or no colocalization of hemozoin and iron staining (black arrow).

Supplemental Table 1 Results of experiments 1, 2, and 3 showing the data of parasitemia, CD14 counting, hematocrit, hemoglobin, rectal temperature, weight and spleen/body weight ratios.
