# Supplementary figures and images for: Increased Plasmodium falciparum Parasitemia in Non-splenectomized Saimiri sciureus Monkeys Treated with Clodronate Liposomes
Source: Front Cell Infect Microbiol. 2017 Sep 21;7:408. doi: 10.3389/fcimb.2017.00408 (PMC5613086; doi:10.3389/fcimb.2017.00408)

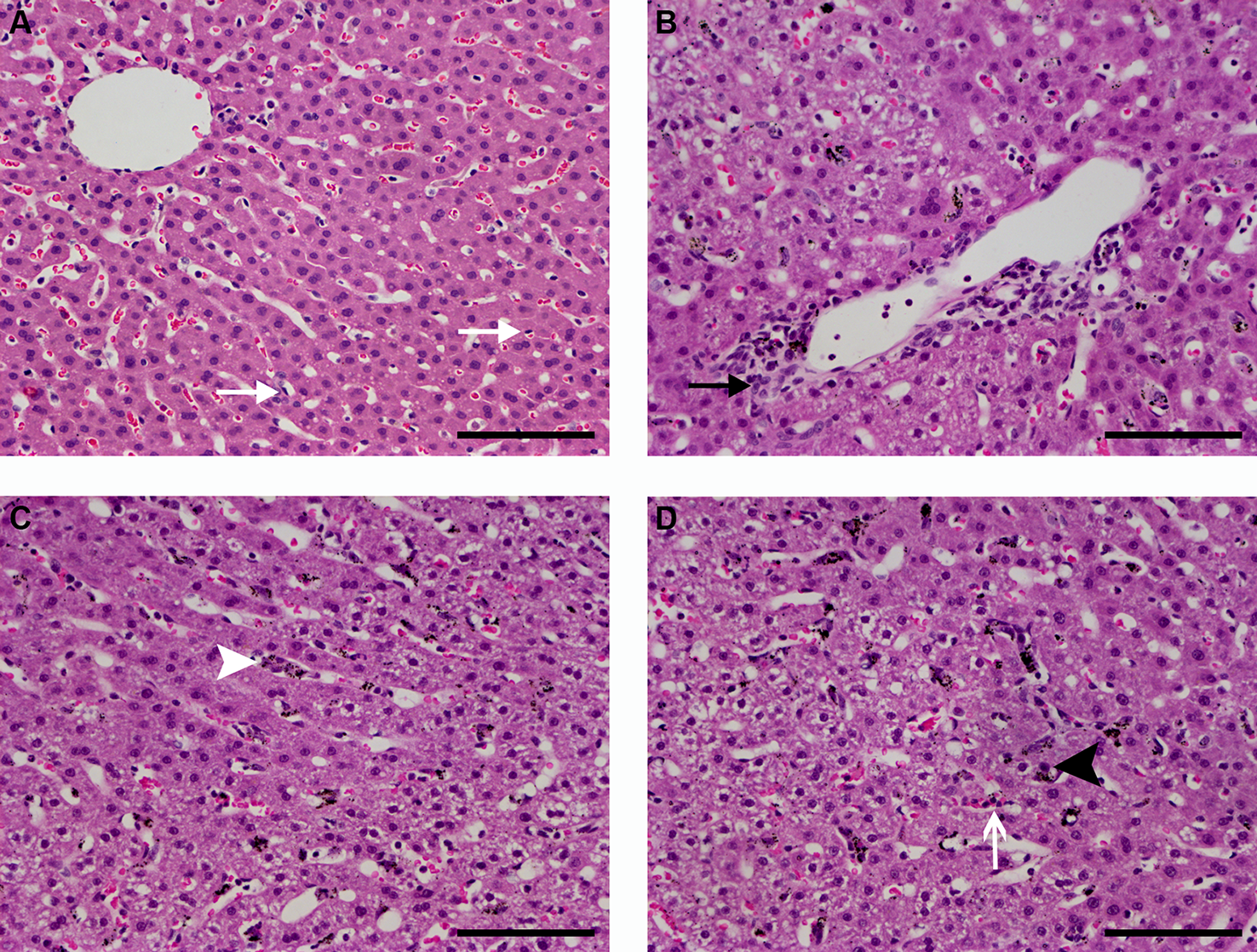

Supplement: Supplemental Figure 1 — Liver sections, HE (bar: 100 μm). (A) Liver of an uninfected, control animal that received PBS (code PA67), in higher magnification than Figure 8A, showing Kupffer cells (arrows). (B–D) Liver of a P. falciparum-infected Saimiri that received PBS (code 141: treated at day 17, with 0.69% parasitemia, killed 5 days later), in greater detail compared to Figure 8C. Portal infiltrates (black arrow), increased cellularity in sinusoids with presence of mononuclear cells (white arrowhead), erythroid cells (thin arrow) and plasma cells (black arrowhead). [file Image1.TIF]

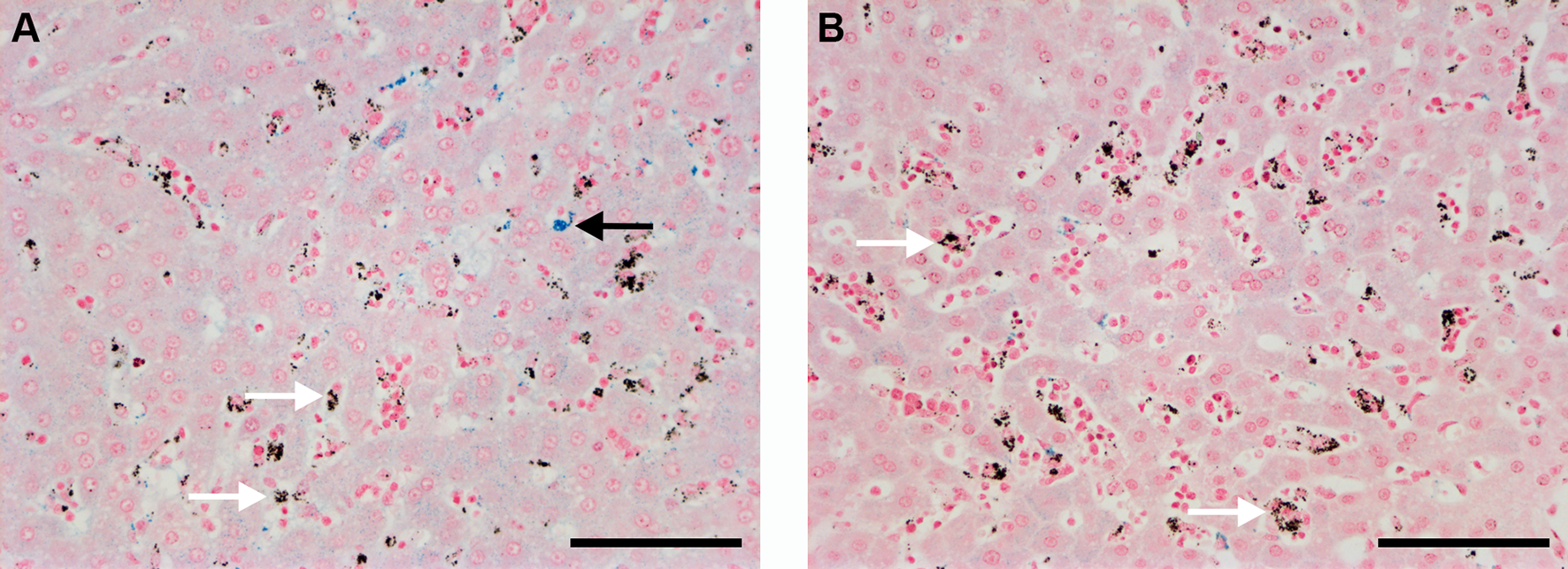

Supplement: Supplemental Figure 2 — Liver sections, Perls (bar: 100 μm). (A,B) Liver of a P. falciparum-infected Saimiri that received PBS (code 216: treated at day 17, with 1.3% parasitemia, killed 5 days later), in greater detail compared to Figure 9C. Intrasinusoidal macrophages, circulating or attached to the sinusoidal wall, laden with malarial pigment (white arrows). There was little or no colocalization of hemozoin and iron staining (black arrow). [file Image2.TIF]
